# High-density carrier-accumulated and electrically stable oxide thin-film transistors from ion-gel gate dielectric

**DOI:** 10.1038/srep18168

**Published:** 2015-12-18

**Authors:** Mami N. Fujii, Yasuaki Ishikawa, Kazumoto Miwa, Hiromi Okada, Yukiharu Uraoka, Shimpei Ono

**Affiliations:** 1 Graduate School of Materials Science, Nara Institute of Science and Technology, 8916-5 Takayama, Ikoma, Nara 630-0192, Japan; 2 Central Research Institute of Electric Power Industry 2-11-2, Iwado-kita, Komae, Tokyo 201-8511, Japan

## Abstract

The use of indium–gallium–zinc oxide (IGZO) has paved the way for high-resolution uniform displays or integrated circuits with transparent and flexible devices. However, achieving highly reliable devices that use IGZO for low-temperature processes remains a technological challenge. We propose the use of IGZO thin-film transistors (TFTs) with an ionic-liquid gate dielectric in order to achieve high-density carrier-accumulated IGZO TFTs with high reliability, and we discuss a distinctive mechanism for the degradation of this organic–inorganic hybrid device under long-term electrical stress. Our results demonstrated that an ionic liquid or gel gate dielectric provides highly reliable and low-voltage operation with IGZO TFTs. Furthermore, high-density carrier accumulation helps improve the TFT characteristics and reliability, and it is highly relevant to the electronic phase control of oxide materials and the degradation mechanism for organic–inorganic hybrid devices.

Transparent amorphous oxide semiconductor thin-film transistors (TFTs) have been widely investigated with the goal of optimally exploiting their low leakage current and transparency^1^. In particular, TFTs based on amorphous indium–gallium–zinc oxide (IGZO) have demonstrated a field-effect mobility as high as 10 cm^2^/(V∙s), which exceeds that of amorphous silicon TFTs^1^. Although IGZO TFTs are produced by using a low-temperature process, they provide high field-effect mobility (*μ*_FE_) despite the amorphous phase induced under low-temperature deposition^2^. Therefore, the use of IGZO has paved the way for high-resolution uniform displays or integrated circuits with transparent and flexible devices. However, achieving highly reliable devices using IGZO in a low-temperature process remains a technological challenge because IGZO deteriorates under the influence of impurities in the atmosphere or the electrical stress of applied voltages.

In order to achieve highly reliable long-term device operation, some research groups have examined inorganic gate insulators and passivation films layered on IGZO thin films, which are fabricated using a high-temperature process^3^,^4^. However, IGZO TFTs have demonstrated rather poor characteristics and a degradation from their initial performance as a result of electron trap or carrier generation due to the adsorption of oxidation or hydrogen from the atmosphere^5^,^6^. To avoid these problems, a high-quality passivation film on the channel has been considered in order to reduce oxidation and hydrogen adsorption. In addition, introducing a top-gate structure to TFTs with high-quality gate insulators can address these issues because the gate insulator film acts as a passivation film. In order to further optimise the devices, numerous combinations of semiconductors and dielectrics are being extensively tested because the interfacial phenomena are crucial to determining their long-term stability. However, IGZO TFTs still have the problem of threshold voltage (*V*_th_) shifts that result from the electrical stress caused by the applied voltage^7^,^8^.

Furthermore, because a low-voltage operation allows activation under a low electrical field in the channel material and at the interface between the channel and gate insulator, ultra-low voltage TFT operation is essential to reduce the electrical stress for highly reliable TFTs because IGZO TFTs are degraded by an applied voltage, and the amount of *V*_th_ shift increases with the applied voltage. Achieving a high current with low-voltage operation provides a strong advantage for driving organic light-emitting diode (OLED) displays that operate on a current. For low-voltage operation, insulating materials that can accumulate carriers at a high density in a semiconductor film are necessary. Some studies have demonstrated low-voltage-operation IGZO TFTs with high-capacitance and high-k material gate insulators for driving OLED devices^9^,^10^,^11^. However, the fabrication of these films is generally difficult at temperatures below 150 °C because the gate-leakage current is rather large.

Recently, transistors that employ an electric double layer have been realised by using ionic liquid or electrolytes^12^,^13^,^14^,^15^. For example, given an oxide semiconductor and ionic liquid combination, some studies have demonstrated high-density carrier accumulation in the channel^16^,^17^. Introducing an ionic liquid enables carrier accumulation in the channel to high densities of up to 10^14^ cm^−3^, which enables operation at an ultra-low voltage^18^. However, the reliability under long-term electrical stress in an organic–inorganic combination has not been discussed. Moreover, choosing a proper ionic liquid can counter the effect of hydrogen on IGZO TFTs, which have top-gate structures, because of the presence of some hydrophobic ionic-liquid materials^19^,^20^,^21^. Therefore, determining the electrical stress reliability of IGZO TFTs by using an ionic-liquid gate dielectric has important implications for the application of organic–inorganic hybrid devices.

In this letter, we present the application of IGZO TFTs with an ionic-liquid gate dielectric in order to achieve high reliability, and we discuss a mechanism for the device degradation of this organic–inorganic hybrid device under long-term electrical stress.

## Results

**Figure 1:**
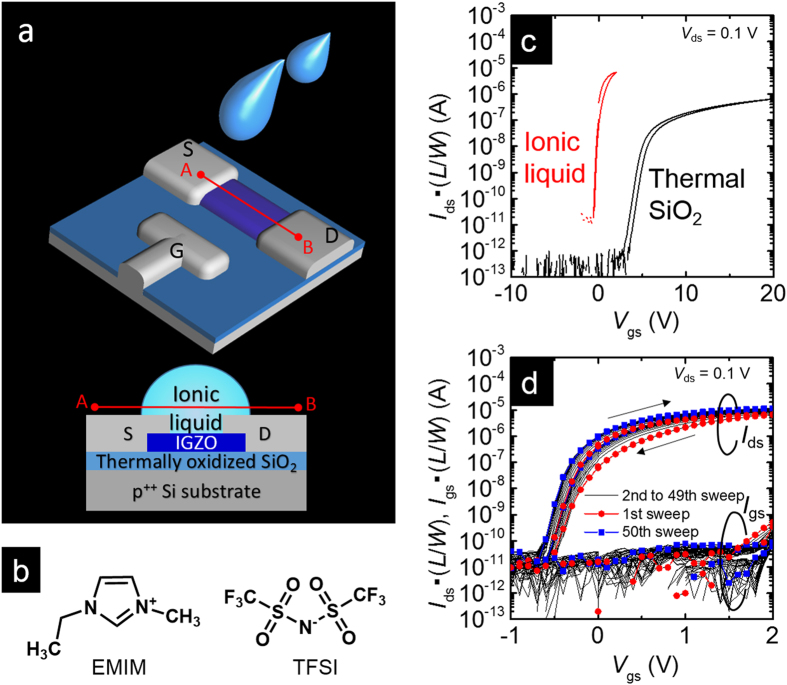
Device structures and characteristics of the IGZO TFT with ionic liquid used in this study. **(a)** Image and schematic cross-sectional view of the IGZO TFT with ionic liquid. The underside image is the cross-sectional cut between points A and B, which are shown in the upper TFT image. **(b)** EMIM-TFSI ionic liquid structure. **(c)** Transfer characteristics and hysteresis curve of the IGZO TFTs with ionic liquid and SiO_2_ gate insulators. **(d)** Fifty-cycle measurement of the IGZO TFTs with ionic-liquid gate dielectric and gate leakage current, where the maximum values of *V*_gs_ are 2 and 20 V for the ionic-liquid and thermal SiO_2_ gate insulator TFTs, respectively, and *V*_ds_ is 0.1 V. The applied *V*_gs_ swept from 0 V toward a negative voltage, returned to 0 V, swept toward a positive voltage, and returned again to 0 V.

Figure 1a displays the image and schematic cross-sectional view of the IGZO TFT used in this study. The underside is shown and represents the cross-sectional image cut between points A and B in the upper TFT image. The image explains the structure of the source-drain electrode and IGZO channel. The highly hydrophobic and highly stable ionic liquid of 1-ethyl-3-methylimidazolium bis(trifluoromethylsulfonyl)imide (EMIM-TFSI), which is shown in Fig. 1b, was used in our experiment. The highly doped Si substrate can act as a gate electrode when driven by the bottom gate, which has a SiO_2_ gate insulator structure. The platinum-covered molybdenum top-gate electrode was applied to the top-side-gate structure with an ionic-liquid gate dielectric, which allowed us to examine the liquid-gate and solid-gate TFT performances on the same substrate. The distance between IGZO and the Mo-gate electrode was 30 μm.

Our selected combination produced excellent TFT characteristics, which are presented in Fig. 1c (red curve). The TFTs that combined the EMIM-TFSI gate dielectric and IGZO channel provided a substantially high drain current (*I*_ds_) of ~10^−6^ A at a gate voltage (*V*_gs_) of 2 V and a significantly small subthreshold swing (*SS*) of 97 ± 25 mV/decade, even when the applied drain voltage (*V*_ds_) was 0.1 V. Here, *I*_ds_ was proofed by using the channel width (*W*) and length (*L*) as *I*_ds_ × *L*/*W*. The threshold voltage (*V*_th_) was approximately zero. On the other hand, the TFT with a thermal SiO_2_ gate insulator demonstrated a lesser value for *I*_ds_ (~10^−7^ A) when *V*_gs_ = 20 V and a large *SS* of 420 ± 18 mV/decade; this is represented by the black curve in Fig. 1c. Table 1 lists the obtained data of the average values for 10 measurements. These parameters were calculated from using the measurements provided by biasing *V*_gs_ from 2 V to −2 V. The variations in *V*_th_ and *SS* (*V*_th-var_ and *SS*_var_) over 50 cycles of transfer curves of the ionic-liquid gate IGZO TFT were exceedingly small: *V*_th-var_ = −0.1 V and *SS*_var_ = −45 mV/decade, as shown in Fig 1d. Although this is currently under study, these variations were most likely due to the carrier generation or filling of the trap states with carriers that were accumulated by the ionic liquid. The relatively large value for *I*_ds_ in the off region resulted from the involvement of the ion motions in the ionic liquid, which suggests that a further reduction of *I*_ds_ in the off region should be expected. These results clearly indicate low-voltage operation with an extremely small *SS*, which was achieved by introducing the EMIM-TFSI ionic liquid.

**Figure 2:**
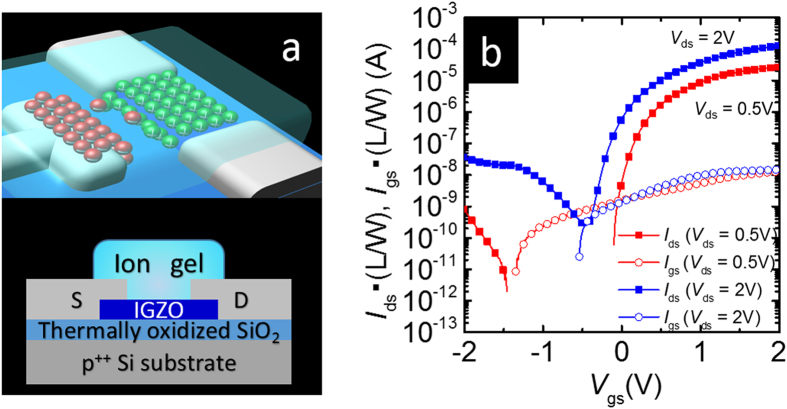
Device structures and characteristics of the IGZO TFT with an ion gel. **(a)** Image and schematic cross-sectional view of the IGZO TFT with an ion gel. The underside image is the cross-sectional cut, which is shown in the upper TFT image. **(b)** Transfer characteristics and gate leakage current of the IGZO TFTs with an ion gel.

Because the liquid was not in an improper material phase during either the device fabrication process or reliability study, we gelated it as shown in Fig. 2a. Note that the ion gel could maintain a high capacitance of above 1 μF/cm^2^ until a frequency of approximately 1 kHz was achieved, which is demonstrated in Fig. S1 of the online supplement. Figure 2b shows the IGZO TFT characteristics when using an ion-gel gate dielectric. Given the evidence of its effectiveness, we concluded that the ion gel can be applied to devices.

**Figure 3:**
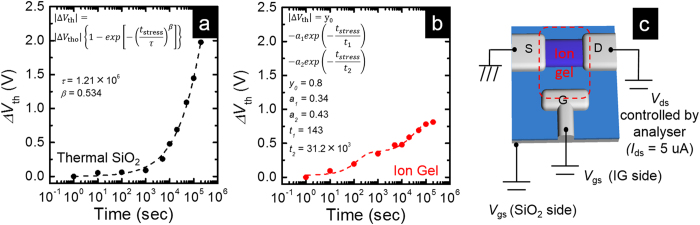
*V*_th_ shifts (*△V*_th_) under a long-term current stress according to the stress time. (**a**)△*V*_th_ in the IGZO TFT with a thermal SiO_2_ gate insulator and (**b**) with an ion-gel gate dielectric. The applied gate stress voltages were *V*_gs_ = 10 V for the SiO_2_ gate dielectric and *V*_gs_ = 2 V for the ion-gel gate dielectric. The applied drain voltages were fixed at *I*_ds_ = 5 μA. The symbols indicate the measurement results, and the broken lines represent the results calculated using (**a**) the stretched exponential equation and (**b**) the two exponential equations. (**c**) Image of the device and the measurement method of the reliability.

Figure 3 presents the *V*_th_ shift (∆*V*_th_) results under a long-term current stress; Fig. 3a shows the bottom gate, and Fig. 3b shows the ion-gel applied top gate. The drain voltages were controlled in order to maintain *I*_ds_ = 5 μA by using a semiconductor parameter analyser (Agilent B1500A), as shown in Fig. 3c. The gate stress voltages were *V*_gs_ = 10 V for the SiO_2_ gate dielectric structure and *V*_gs_ = 2 V in the ion-gel gate dielectric structure. The symbols indicate our measured results, and the broken lines signify the results that were calculated by using Equations 1 and 2. These measurements were taken in air.

## Discussion

The carrier’s excitation and recombination mechanisms for amorphous semiconductor materials can be expressed in a stretched exponential equation. Lee *et al.* confirmed that the IGZO TFT degradation follows this relation^22^. Given a SiO_2_ dielectric, *V*_th_ degradation occurs according to the following stretched exponential equation^23^:


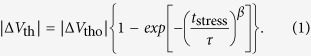


Here, *△V*_th0_ represents the *V*_th_ shift amount as the time approaches infinity; this is provided by *V*_g-stress_ – *V*_th-initial_. *V*_g-stress_ is the applied stress voltage, *V*_th-initial_ is the initial *V*_th_ voltage before the stress test, *t*_stress_ is the stress time, *τ* is the characteristic trapping time for the carriers, and *β* is the stretched exponential exponent. In this case, which is shown in Fig. 3a, the measured values for *τ* and *β* were 1.21 × 10^6^ s and 0.536, respectively. When the stress time was 2 × 10^5^ s, *△V*_th_ reached approximately 2 V. This equation is commonly used for the relaxation process of the random system. In particular, it can be expressed as a temperature-dependent model of the energy transmission or as an Urbach tail caused by dipole interaction when *β * = 0.5^24^,^25^. This model is understood to represent the carrier trapping that occurs in the TFTs^23^. Therefore, degradation of the IGZO TFTs with an SiO_2_ gate insulator results from electron trapping, which is related to semiconductor and insulator films and is caused by electrical stress.

On the other hand, IGZO TFTs with an ion-gel gate dielectric exhibited markedly different behaviour. We can expect stability with ion-gel gate IGZO TFTs when compared with a TFT that uses a conventional SiO_2_ gate insulator. Therefore, we successfully fitted the *V*_th_ degradation by using Equation 2 rather than the stretched exponential equation:


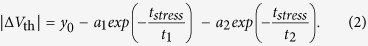


Figure 3b shows the result of Equation 2, which includes two exponential equations. There were two inflection points at approximate stress times of 10^3^ and 10^5 ^s. Here, the decay constants *t*_1_ and *t*_2_ were 143 s and 31.2 ks, respectively. They indicate that the degradation mechanism included slow and fast reactions rather than an ionic charge^26^. Therefore, this degradation mechanism cannot be addressed by using the previously discussed conventional mechanism. We assumed that the *V*_th_ shift degradation depends on the progress of the chemical reaction, which is related to the ionic liquid, i.e. the oxidation or hydrogen adsorption of the ion gel or the melting reaction of metal atoms by the ion gel, in addition to the carrier trapping of the IGZO. This implies that the *V*_th_ shift in the ion-gel gated TFT was primarily produced by the breaking of the ion gel or chemical reaction of the ion gel with IGZO and not because of the electrical trapping related to IGZO films. The problem of stability remains when using an ionic liquid or gel because these incorporate oxygen or water when stored under atmosphere. Thus, an ion-gel gate IGZO TFT with lamination to protect it from oxygen and water produced a remarkably stable and low-voltage operation device. Although further study on the degradation mechanism is necessary, the ion-gel gate IGZO TFT exhibited extremely stable characteristics.

In summary, we successfully obtained low-voltage IGZO TFTs by using an ionic-liquid gate dielectric, especially when compared with bottom-gate IGZO TFTs that use SiO_2_ gate insulators. Ionic-liquid gate IGZO TFTs also produced extremely small *SS* values near the theoretical limit and an on-state current that was an order of magnitude greater than that of conventional SiO_2_ gate IGZO TFTs, even when derived under a voltage of 2 V. In other words, high-density carrier accumulation was achieved in the IGZO channel using a liquid gate dielectric. Moreover, the ion-gel gate IGZO TFTs exhibited excellent reliability under electrical stress when compared with the IGZO TFTs that used SiO_2_ gate insulators, even when measured in air. Therefore, the ion gel is a promising insulating material for the low-temperature fabrication of IGZO TFTs and is one solution to achieving flexible IGZO TFTs. Our results explicitly demonstrate that an ionic liquid or gel gate dielectric provides highly reliable and low-voltage IGZO TFTs. The high-density carrier accumulation helps improve TFT characteristics and reliability, and it is highly relevant to discussions of the electronic phase control of oxide materials and degradation mechanism for organic–inorganic hybrid devices.

## Methods

We fabricated a top-gate type IGZO TFT with ionic liquid, as shown in Fig. 1a. We then examined the transfer characteristics of TFTs and their stability under electrical stress. The fabricated TFT can also serve as a bottom-gate type TFT. IGZO films with stoichiometric ratios of 2:2:1:7 and a thickness of 70 nm were deposited at room temperature by using radio frequency (RF) magnetron sputtering on thermally oxidised 100-nm-thick SiO_2_, which was grown on a highly doped p^++^ Si substrate (<0.01 Ω∙cm) and patterned with wet etching. Platinum-covered molybdenum electrodes, which were used as the source, drain, and gate electrodes, were prepared by using RF magnetron sputtering and the lift-off technique. The annealing process, which was performed in air at 300 °C for 2 h, completed the electrode fabrication. Next, we drop-casted the ionic liquid on the TFT channel region.

For ionic liquid gelation, we prepared the solution of ionic liquid with polymer by melting the polymer DM37-M06 (DAISO Co., Ltd.) with acetonitrile solvent and mixing it with the ionic liquid, a cross-linking agent, and a photo-polymerisation initiator (Benzophenon). Here, the mixing ratio of the polymer and ionic liquid was 1:1. After this solution was drop-casted on the channel, the substrates were baked at 100 °C for 5 min and irradiated at an ultraviolet wavelength of 254 nm for 10 min.

To measure the initial characteristics of the transistor, we used Agilent B1500A in a nitrogen ambient at room temperature at a scanning speed of 0.065 V/s by biasing from *V*_gs_ = 0 V to 2 V. The bias was turned to 0 V and swept to −2 V before being turned to 0 V again. Then, we checked the reliability under normal conditions by measuring the TFT characteristics with Agilent B1500A in air at room temperature. The characteristics were measured before and after the current stress each time.

We measured the capacitance of the ionic liquid and its gel by using the AC impedance technique (Solartron 1260 and 1296). We formed a well structure of polydimethylsiloxane (PDMS) and deposited the Au film on the PDMS surface. We then drop-casted the ionic liquid onto the well on the Au metal. Au-coated PDMS was placed in the ionic liquid, and an Au/ionic liquid/Au structure was formed. In the IG case, the Au metal was deposited on the Si substrate and placed on the gel-sheet, and Au was deposited again on the gel-sheet. Here, the gap thickness between Au electrodes was 25 μm. The structure of the samples is shown in Fig. S1b and c of the online supplement. We also performed the measurement by using a TFT structure as shown in Fig. S2b. The capacitance of the ion gel showed a frequency dependence and was 3.6 μF/cm^2^ at 10 Hz, as shown in Fig. S2a of the online supplement.

## Additional Information

**How to cite this article**: Fujii, M. N. *et al.* High-density carrier-accumulated and electrically stable oxide thin-film transistors from ion-gel gate dielectric. *Sci. Rep.* **5**, 18168; doi: 10.1038/srep18168 (2015).

## Supplementary Material

Supplementary Information

## Figures and Tables

**Table 1:** TFT parameters of ionic-liquid and SiO_2_ gate TFTs.

**Gate insulator**	**Maximum *I*_ds_ · (*L*/*W*) (A)**	***SS* (mV/dec)**	***V*_th_ (V)**
ionic liquid	~10^−6^ at V_gs_ = 2 V	97 ± 25	0.66 ± 0.26
SiO_2_	~10^−7^ at V_gs_ = 20 V	420 ± 18	5.12 ± 1.47

The average values of 10 measurements are given for each gate insulator.
