# Supplementary material for: High-density carrier-accumulated and electrically stable oxide thin-film transistors from ion-gel gate dielectric
Source: Sci Rep. 2015 Dec 18;5:18168. doi: 10.1038/srep18168 (PMC4683535; doi:10.1038/srep18168)
Supplement: Supplementary Information [file srep18168-s1.pdf]

Supplimentary Information

# High-density carrier-accumulated and electrically stable oxide thin-film transistors from ion-gel gate dielectric

*Mami N. Fujii*, Yasuaki Ishikawa*, Kazumoto Miwa, Hiromi Okada, Yukiharu Uraoka, and Shimpei Ono*

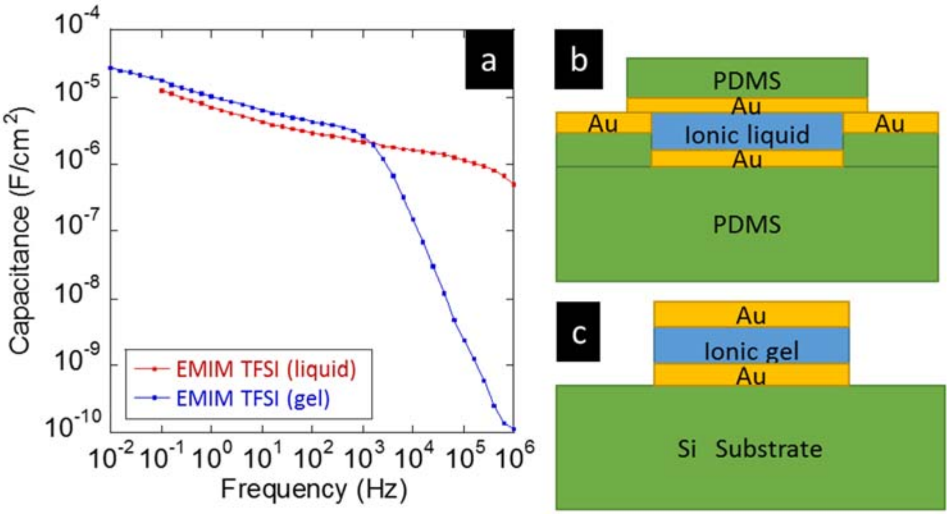


**Figure S1.** (a) Capacitance according to the frequency of EMIM-TFSI ionic liquid and its gel. (b) and (c) Structure of capacitance measurement using ionic liquid (b) and ion gel (c).

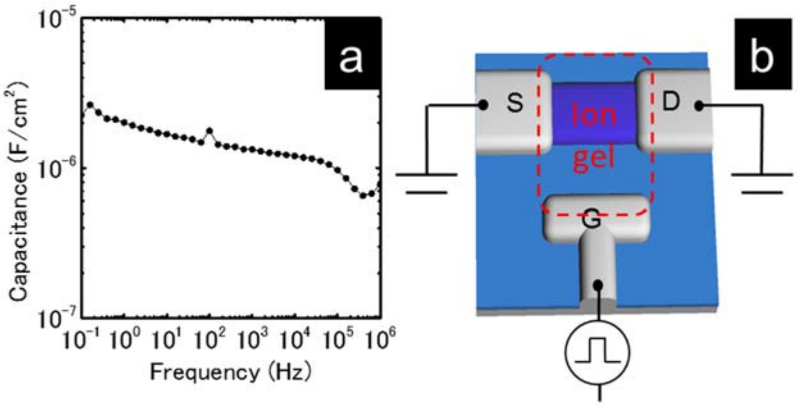


**Figure S2.** (a) Capacitance according to the frequency of EMIM-TFSI ionic liquid. (b) Structure of capacitance measurement using IGZO TFT with ionic liquid.
